# Metaomics in Clinical Laboratory: Potential Driving Force for Innovative Disease Diagnosis

**DOI:** 10.3389/fmicb.2022.883734

**Published:** 2022-06-17

**Authors:** Liang Wang, Fen Li, Bin Gu, Pengfei Qu, Qinghua Liu, Junjiao Wang, Jiawei Tang, Shubin Cai, Qi Zhao, Zhong Ming

**Affiliations:** ^1^Department of Bioinformatics, School of Medical Informatics and Engineering, Xuzhou Medical University, Xuzhou, China; ^2^Department of Laboratory Medicine, Huaiyin Hospital, Huai’an, China; ^3^The First School of Clinical Medicine, Xuzhou Medical University, Xuzhou, China; ^4^State Key Laboratory of Quality Research in Chinese Medicines, Macau University of Science and Technology, Taipa, Macao SAR, China; ^5^College of Computer Science and Software Engineering, Shenzhen University, Shenzhen, China; ^6^School of Computer Science and Software Engineering, University of Science and Technology Liaoning, Anshan, China

**Keywords:** microbiology, microbiome, omics, biomarker, diseases, rapid diagnosis

## Abstract

Currently, more and more studies suggested that reductionism was lack of holistic and integrative view of biological processes, leading to limited understanding of complex systems like microbiota and the associated diseases. In fact, microbes are rarely present in individuals but normally live in complex multispecies communities. With the recent development of a variety of metaomics techniques, microbes could be dissected dynamically in both temporal and spatial scales. Therefore, in-depth understanding of human microbiome from different aspects such as genomes, transcriptomes, proteomes, and metabolomes could provide novel insights into their functional roles, which also holds the potential in making them diagnostic biomarkers in many human diseases, though there is still a huge gap to fill for the purpose. In this mini-review, we went through the frontlines of the metaomics techniques and explored their potential applications in clinical diagnoses of human diseases, e.g., infectious diseases, through which we concluded that novel diagnostic methods based on human microbiomes shall be achieved in the near future, while the limitations of these techniques such as standard procedures and computational challenges for rapid and accurate analysis of metaomics data in clinical settings were also examined.

## Introduction

**FIGURE 1:**
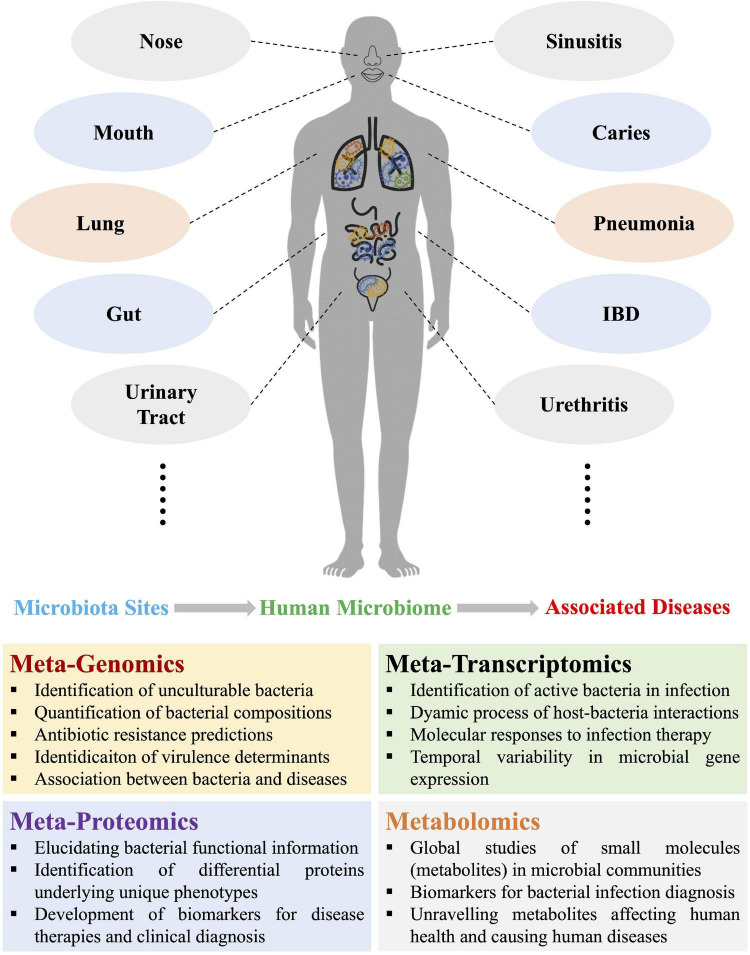
Schematic illustration of the four omics approaches used in current and potential studies of human microbiomes and the associated diseases due to microbiota dysbiosis, which mainly involves metagenomics, metatranscriptomics, metaproteomics, and metabolomics. Representative functions of each of the four metaomics techniques were also listed. IBD, inflammatory bowel disease.

In recent years, human microbiome studies revealed that dysbiosis of microbial communities could lead to dysfunction of host machineries, causing a broad spectrum of diseases (Wang et al., 2017; Kho and Lal, 2018). Thus, understanding the associations of particular bacterial species with diseases could hold the potential of providing new treatment targets and therapeutic approaches in clinical settings (Almeida et al., 2019). Until the last two decades, conventional methods such as bacterial culture and biochemical tests were normally considered as gold standards of bacterial diagnosis and widely employed in clinical laboratory (Wang et al., 2021). Driven by the technological developments and economic benefits, molecular methods such as PCR and immunoassay are gradually becoming available and popular for bacterial diagnosis. However, both conventional microbiology and novel molecular techniques only satisfy with the simplicity and controllability of the reductionism framework by focusing on limited number of genes and bacterial species. Although the reductionist approach could reveal the individual genetics and physiology, contributing to the understanding of complex microbial behaviors in nature (Tecon et al., 2019), these observations and conclusions are difficult to be directly applied to the physiology of whole ecological systems like human-microbiota interactions (Fang et al., 2011). Around a decade ago, microbiome was merely a word that was mainly heard of by fellow scientists and the public was rarely familiar with the concept. With the recent rise of microbiome research, more and more studies acknowledge that microbes work together as a community to achieve key functions related with various aspects of human health ranging from metabolic disease to gastrointestinal disorders to emotional disturbance. A variety of techniques have been developed so far to dissect the human microbial communities in common niches such as mouth, gut, vagina, etc. both spatially and temporally, which include metagenomics, metatranscriptomics, metaproteomics, and metabolomics (Figure 1). These techniques are also known as metaomics when combined for integrated analysis. In addition, both the public and popular press show more and more interests in this novel field (Marcon et al., 2021), which lies the ground for metaomics to be developed and accepted as innovative bacterial diagnostics tools.

Metaomics is an innovative integration approach that is based on the in-depth analysis of human microbiomes, which has spurred a paradigm shift in understanding human health and detecting infectious diseases (Xu and Yang, 2021). Apparent advantages have been reported that makes these techniques with promising potentials in clinical diagnosis of bacterial infections, such as quantification of bacterial compositions, detection of unculturable bacterial pathogens, profiling of bacterial antibiotics-resistant genes, identification of virulence factors in large scale, and establishment of associations between bacteria and diseases, etc., all of which could be realized through metagenomic analysis (Wang et al., 2022). In addition, the dynamics of microbe–microbe interplays, host–microbe interactions, energy metabolism, and chemical cycling during bacterial infection could be elucidated through metatranscriptomic studies, which could not only improve the understanding of bacterial pathogenicity, but also facilitate biomarker discovery and development of microbial therapeutics (Zhang et al., 2021). Moreover, metatranscriptomics is also able to identify active bacteria and temporal variability of bacterial gene expressions during infection. Metaproteomics focuses on the dynamic changes of whole proteins in specified microbial communities, which could not only obtain functional information of bacterial communities, but be also able to link genes (proteins) with underlying phenotypes, which could also contribute to the development of biomarkers for therapies and diagnosis. As for metabolomics, it is a community overview of individual microbial metabolism, which focuses on global profiles of metabolites (small molecules), aiming to reveal biomarkers for bacterial infection diagnosis and also unravel metabolites concerning human health. Due to the complex interplays between metabolites during microbe-microbe and microbe-host interactions, metabolic networks based on constraint-based reconstruction and analysis (COBRA) and genomic-scale metabolic models (GEMs) are frequently constructed so as to understand microbiome–metabolome links and facilitate the translation of the findings into effective and novel therapeutics (Heinken et al., 2021; Jansma and El Aidy, 2021).

Although metaomics studies are increasingly wide-spreading and are presumed to be novel diagnostic tools in clinical laboratory in future, these techniques are mainly confined to the research field at current stage due to the disadvantages that are so far hard to overcome, such as high costs for experimental procedures and lack of gold standard for sample collection and data analysis, etc. (Chiu and Miller, 2019; Shakya et al., 2019; Dias et al., 2020). In addition, for metabolomics, techniques with acceptable sensitivity are only just being developed, while computational analysis and integration of metaomics data are other challenges that hinder the potential application of metaomics techniques in clinical settings, though data management and comparative analysis system are actively explored at current stage (Chen et al., 2019; Wang et al., 2022). In this mini-review, we will not look into the technical details of metaomics approaches; in contrast, we endeavor to focus on the application potentials of metaomics techniques for their rapid and accurate diagnosis of bacterial pathogens and infections. However, it should be noted that, in most studies, the presence of certain species, the altered levels of microbes, and the changed abundances of microbial transcripts, proteins or metabolites, have not been proven as causes for diseases but only associations. Therefore, the perspectives for advancing the functional and translational microbiome research in clinical settings, which may also facilitate the implementation of metaomics-based precision medicines, will be discussed in general manner in this mini-review.

## Potential Applications of Metaomics in Clinical Diagnosis

### Metagenomics

In clinical laboratories, many pathogens are unamenable to be cultured or sometimes exist in a viable but non-culturable (VBNC) state, which makes them very difficult to be detected *via* conventional microbiological approaches such as microscopy and biological tests, leading to a great risk to public health (Li et al., 2014). Metagenomic next generation sequencing (mNGS) is the analysis of a collection of genomes from a mixed community of microbial organisms, which can rapidly quantify the organism diversity and microbial composition of a specific microbiota in a timely manner, showing attractive features for clinical diagnosis. It should be emphasized that mNGS (whole shotgun metagenomic sequencing) is not the same as 16S rRNA gene sequencing (16S sequencing) because the single amplicon sequencing cannot be analyzed together with other omics datasets. In particular, 16S sequencing only amplifies portions of the hypervariable regions (V1–V9) of the bacterial 16S rRNA gene, which could lead to potential biases in the representation of the taxonomic units due to the choice of primers (Laudadio et al., 2018). In addition, studies also showed that 16S sequencing only detects a part of gut microbiota community revealed by mNGS (Durazzi et al., 2021). In contrast, the mNGS approach sequences all the DNA materials (viruses, bacteria, fungi, and micro-eukaryotes) in the microbiome rather than just bacteria as found through 16S sequencing, which generated more sequenced reads per sample, hence, higher resolutions in taxonomic assignments at species level and also higher sequencing costs (Peterson et al., 2021).

In a systematic review, Quince et al. (2017) summarized in details of the metagenomic analysis procedures from sample preparations to computational pipelines, which offers a biotechnological promise in therapeutic discovery of human health. In a recent perspective, Segata (2018) emphasized the importance of accurately elucidating human-associated microbial communities at strain level through developing new computational tools, which can link strain variants to host phenotypes and holds the potential of understanding the personalized host–microbiome interactions. In fact, with the fast development of metagenomic techniques, this culture-independent approach has been applied in detecting microbial pathogens in public health (Miller et al., 2013; Chiu and Miller, 2019), identifying genes or genetic mutations conferring resistance to antimicrobial drugs (De, 2019; De Abreu et al., 2021), and enabling genotyping analysis for molecular epidemiology and so on (Robert and Filkins, 2019), which makes the method gradually transiting from research fields to clinical laboratories (Chiu and Miller, 2019) and slowly integrating into clinician’s toolbox to identify infectious diseases (De Vries, 2021), though it functions as a diagnostic tool yet to be widely established due to a variety of issues such as costs, turnaround time, sensitivity, specificity, validation, and reproducibility, etc. in clinical microbiology laboratories.

In specificity, the clinical applications of mNGS involves dissecting healthy microbial compositions in various body parts such as mouth, respiratory tract, gut, central nervous system (CNS), urinary tract, vagina, etc. (Gu et al., 2019) and revealing the aberrant bacterial compositions in various clinical samples such as saliva, bronchoalveolar lavage fluid, cerebrospinal fluid (CSF), urine, vaginal secretion, and other body fluids or infected tissues (Chiu and Miller, 2019), through which abnormal bacterial genera and species could be identified and might be used to serve for potential clinical diagnosis of human infectious diseases such as periodontitis (Curtis et al., 2020), pneumoniae (Thibeault et al., 2021), meningitis (Moir, 2015), urethritis (Srinivasan et al., 2021), vaginosis (Onderdonk et al., 2016), etc. In addition, non-infectious human diseases were also reported to be associated with microbiota dysbiosis. For example, it was identified that during diabetes and cardiovascular disease (CVD), the microbial diversity of blood microbiota is vastly transformed, in which the two bacterial genera *Staphylococcus* and *Klebsiella* were predominant in the blood of patients with type 2 diabetes mellitus (T2DM), while high *Actinobacteria/Proteobacteria* ratio was consistently associated with CVD (Velmurugan et al., 2020). Therefore, these alterations in bacterial compositions hold the promise to be translated into potential indictors for the clinical diagnosis of the two diseases. As for the CNS, it was suggested that no detectable microbial community existed in healthy CSF because blood–brain barrier (BBB) is able to protect against microbial invasions, though such a claim is still controversial due to the difficulties in the identification of contamination (Kang et al., 2021). Recently, under pathological conditions, studies revealed that bacterial pathogen *Porphyromonas gingivalis* was found in the brains of Alzheimer’s disease (AD) patients and CSF of patients with probable AD (Dominy et al., 2019); however, the presence of *P. gingivalis* DNA in CSF serving as a diagnostic marker for AD promising but debatable, which required further explorations. In addition, there are many other cases involving metagenomic analysis confirmed the application potential of the metaomics techniques in clinical diagnosis due to the associations between human diseases and microbiota dysbiosis. In addition, microbiome research also holds the potential to identify microbial species that are causally associated with cancer phenotypes and unravel the underlying mechanisms behind these associations, which could facilitate cancer diagnosis and transform the treatment strategies for patients with cancer (Banerjee et al., 2015). For a brief summary of the representative studies on the associations between diseases and aberrant microbiota, please refer to Table 1. Taken together, metagenomics can serve as a potential driving force for clinical diagnosis of microbial infections and microbiota-dysbiosis-related diseases with personalized patient cares in future, though there is still a huge gap to fill between basic researches and clinical translations. Therefore, different from microbial culture and biochemical testing, there is still a long way for mNGS to go before the technique could become a vital tool in any clinical testing algorithms.

**TABLE 1:** Comparison of healthy and disturbed microbiota that might contribute to the understanding of certain diseases from microbial perspectives.

Organ, tissues, fluids	Healthy microbiota (predominant bacterial genera)	Disturbed microbiota (abundant bacterial genera/species)	Representative human diseases associated with disturbed microbiota	References
Blood	*Achromobacter, Pseudomonas, Serratia, Sphingomonas, Staphylococcus, Corynebacterium, Acinetobacter*	*Staphylococcus, Klebsiella*	Type 2 diabetes mellitus (T2DM)	Velmurugan et al., 2020
		High *Actinobacteria/Proteobacteria* ratio	Cardiovascular disease (CVD)	
Central nervous system (cerebrospinal fluid)	No detectable microbial community	*Porphyromonas gingivalis*	Alzheimer’s disease (AD)	Roos, 2015; Velmurugan et al., 2020; Kang et al., 2021
		*Streptococcus pneumoniae, Neisseria meningitidis*	Meningitis	
		*Staphylococcus aureus*	Spinal epidural abscess	
Gut (feces)	*Ruminococcus, Clostridium, Lactobacillus, Enterococcus, Bacteroides, Prevotella, Bifidobacterium, Escherichia, Akkermansia*	*Enterobacteriaceae*	Inflammatory bowel disease (IBD)	Durack and Lynch, 2019; Velmurugan et al., 2020
		*Bacteroides* spp.	Type 2 diabetes mellitus	
		*Collinsella, Corynebacterium, Lactobacillus*	Behavioral disorders	
		*Faecalibacterium, Akkermansia, Lachnospira*	Atopic asthma	
Lung (bronchoalveolar lavage fluid)	*Prevotella, Streptococcus, Veillonella, Neisseria, Haemophilus, Fusobacterium*	*Staphylococcus, Haemophilus*	Asthma	Faner et al., 2017; Mathieu et al., 2018
		*Staphylococcus aureus, Burkholderia cepacia*	Cystic fibrosis	
Milk	*Staphylococcus, Streptococcus, Corynebacterium, Cutibacterium, Lactobacillus, Lactococcus, Bifidobacterium*	*Lactobacillus iners, Neisseria subflava, Streptococcus lactarius, Streptococcus cristatus, Staphylococcus aureus*	Sub-acute lactational mastitis	Fernández et al., 2020
Mouth (saliva)	*Streptococcus, Veillonella, Granulicatella, Gemella, Actinomyces, Corynebacterium, Rothia, Fusobacterium, Porphyromonas, Prevotella, Capnocytophaga, Neisseria, Haemophilus, Treponema, Lactobacterium, Eikenella, Leptotrichia, Peptostreptococcus, Staphylococcus, Eubacteria, Propionibacterium*	*Prevotella, Fusobacterium*	Periodontitis	Zarco et al., 2012; Willis and Gabaldón, 2020
		*Neisseria, Selenomonas, Propionibacterium*	Dental caries	
		*Veillonella, Atopobium, Prevotella, Leptotrichia*	Rheumatoid arthritis	
Stomach (gastric juice)	*Streptococcus, Prevotella*	*Firmicutes, Fusobacteria*	Gastroesophageal reflux disease (due to the use of proton pump inhibitor)	Ohno and Satoh-Takayama, 2020
Urinary tract (urine)	*Prevotella, Escherichia, Enterococcus, Streptococcus, Citrobacter*	*Herbaspirillum, Porphyrobacter, Bacteroides*	Urothelial carcinoma	Perez-Carrasco et al., 2021
Vagina (vaginal secretion)	*Lactobacillus* spp., *Actinobacteria, Prevotella, Veillonellaceae, Streptococcus, Proteobacteria, Bifidobacteriaceae, Bacteroides, Burkholderiales*	*Gardnerella, Prevotella, Atopobium, Mobiluncus, Bifidobacterium, Sneathia, Leptotrichia*	Bacterial vaginosis	Chen et al., 2021

*It should be emphasized that the presence of certain species has not been proven as causes for diseases but only associations in most studies. Therefore, we only discuss the possibilities for mNGS method in clinical diagnosis of human diseases through the composition of bacteria in disturbed microbiota, rather than confirming the real applications of the mNGS methods in clinical settings.*

### Metatranscriptomics

Different from metagenomic analysis that focused on the study of taxonomical profiles and microbial compositions in human samples, metatranscriptomics aims to elucidate the functional profiles of metagenomes that inform of the genes that are expressed by the community as a whole under specific conditions, leading to the dynamic understanding of functional ecology of human microbiome (Franzosa et al., 2014; Aguiar-Pulido et al., 2016; Shakya et al., 2019). In addition, during certain circumstances, no linkages between microbiome and diseases could be found at metagenomic level, while correlations at metatranscriptome level could be established. For example, Feng et al. (2019) recruited both metagenomic and metatranscriptomic analyses to dissect the human prostate microbiota from patients with prostate cancer, through which the study revealed that the bacterial composition was not significantly changed between tumor and adjacent benign tissue while gene expression profiles of *Pseudomonas* may be related with metastasis. In fact, with the emergence of the novel notion that microbial associations with certain diseases like oral cancer are actually at functional level of microbial communities rather than at microbial compositional level (Banavar et al., 2021), more and more studies implemented metatranscriptomics or combined metagenomics with metatranscriptomics to determine gene expressions and regulations when the microbiota responded to certain conditions or in certain abnormal states in order to gain comprehensive and functional understandings of human microbiomes (Shakya et al., 2019). Interestingly, metatranscriptomic profiles were more individualized than metagenomic profiles, which had less variable when compared with microbial compositions (Franzosa et al., 2014; Abu-Ali et al., 2018). Currently, many studies have taken the advantages of metatranscriptomics and aimed to elucidate the dynamic gene expressions in the study of human microbiota. For example, Banavar et al. (2021) used both metagenomics and metatranscriptomics to characterize salivary microbiota, which discovered relative abundance of specific bacterial species and gene expressions associated with periodontitis and dental caries. Thus, theses bacterial species and active genes might be possible for evaluating saliva for potential periodontitis and dental caries at pre-clinical stages. Another example using metatranscriptome to study lung cancer patients found that the active presence of two bacteria, *Bacillus megaterium* and *Mycobacterium franklinii*, might play an important role in the occurrence of lung cancer tumors (Chang et al., 2021), which confirmed the potential of metatranscriptomics in identifying the dynamic interactions between microbes and human host in terms of disease progression and severity. It was also recently reported that metatranscriptomics was able to assess the clearance of burn wound infection through differentiating between live and dead organisms and understanding rapid microbial alterations in complex host-microbe samples (Ojala et al., 2021). A variety of other human diseases were also investigated through metatranscriptomics like bacterial vaginosis (Ravel et al., 2013), which could facilitate the identification of the most metabolically active species present in the patients with particular diseases. Therefore, metatranscriptomics is an integral part of the metaomics toward a system level understanding the dynamics of human microbiome in responses to diverse factors.

### Metaproteomics

All the proteins in a microbial community are termed as a metaproteome while the study of taxonomic and functional composition of a microbiota through overall identification of proteins using mass spectrometry is terms as metaproteomics, which is a crucial approach to understand microbial functions in communities (Heyer et al., 2019). Due to its direct insights into microbial phenotypes on large-scale molecular levels, metaproteomics is also a promising tool for clinical diagnostics of human diseases. For example, Long et al. investigated the pathogenesis of colorectal cancer (CRC) through the quantitative comparisons of microbial protein abundances between the CRC patients and the healthy volunteers, which identified 341 significantly different proteins that may serve as biomarkers for distinguishing pathological states and showed that metaproteomics had great value for guiding clinical diagnosis in the future (Long et al., 2020). In addition, a recent in-depth investigation studied the functional compositions of gut microbiota and proteins in a set of fecal samples (treatment-naïve type 2 diabetic, *n* = 77; pre-diabetic, *n* = 80; and normal glucose tolerant, *n* = 97); through a combination of metagenomics and metaproteomics, distinct gut metagenomics and metaproteomics signatures in prediabetics and treatment-naïve type 2 diabetics were discovered, leading to the potential translation of microbiota features into clinical diagnosis biomarkers (Zhong et al., 2019). Previously, Lassek et al. (2015) also used metaproteomics approach to explore the interactions between host and pathogens during catheter-associated urinary tract infections (UTI), which revealed that the asymptomatic phase of catheter-associated UTI could be due to the well-maintained balance of protein levels between bacterial virulence factors and human immune system. Therefore, metaproteomics is also able to elucidate the potential molecular mechanisms of clinical problems. However, so far, the clinical application of quantitative metaproteomics is still in its infancy because of methodological limitations in sample preparations and computational analyses, etc. For example, the great heterogenicity of microbial proteins in any clinical sample significantly hinders the analysis and interpretation of the metaproteome result; in addition, it is also computationally challenging to integrate metaproteomic data with clinical data sets in order to gain clinically meaningful explanations (Blackburn and Martens, 2016). To sum up, further technical developments and innovations are required to facilitate the progress of this promising field.

### Metabolomics

Metabolomics is an analytical technique for the study of metabolic networks by examining the overall changes of small metabolites in biological systems (Wang et al., 2019). As for metabolomics in the study of microbiota, it is a recently emerged application for determining all the metabolites released by microbiomes. Thus, it is a community-based version of single microbial metabolomics in a particular physiological state, which is also known as community metabolomics or environmental metabolomics. In clinical settings, metabolomics could solve the questions like what metabolites are produced under different conditions by the microbiome. In addition, metabolites released by microbial communities normally have responsibilities for the human health that they inhabit, which makes them eligible to serve as biomarkers for clinical diagnosis. In fact, the molecular mechanisms behind how human microbiomes in different body parts correlated with the dynamic alterations of metabolites and causing diseases are starting to be elucidated, which could contribute to the development of preventive and treatment strategies for complex human diseases (Lee-Sarwar et al., 2020). For example, Jansson et al. recruited Ion Cyclotron Resonance Fourier Transform Mass Spectrometry (ICR-FT/MS) to study the causes and etiology of Crohn’s disease (CD) *via* fecal samples from 17 identical twins that were with and without CD, respectively (Jansson et al., 2009). According to the study, the non-targeted metabolic profiling revealed metabolic biomarkers of CD that might serve as diagnostic aims or monitoring tools for CD therapy and prevention (Jansson et al., 2009). In addition, a comprehensive study conducted by Walker et al. (2014) revealed obesity-related metabolite profiles in two different C57BL/6 mouse strains, C57J and C57N, which identified new factors that might be responsible for high-fat diet induced obesity, providing potential new strategy for obesity diagnosis and treatment. In addition, Han et al. (2021) recently developed a novel metabolomics pipeline, which provided a powerful tool for characterizing microorganisms and deciphering the interactions between microorganisms and their host in terms of small metabolites. Although metabolomics is powerful technique and is sensitive enough in profiling metabolites in batch, due to the complex interplays between metabolites, there are still many limitations for its robust applications in clinical settings, which should be addressed and solved during the continuing development and in-depth application of the technique.

### Integration of Metaomics Techniques

**FIGURE 2:**
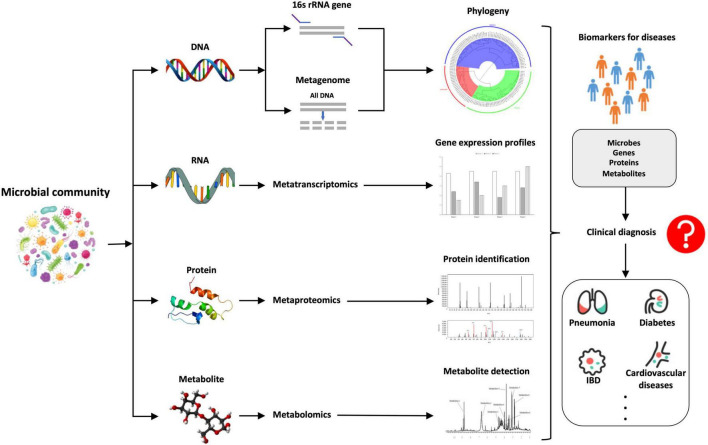
A brief summary of the comparative illustration of the integration of the four metaomics approaches, that is, metagenomics (DNA), metatranscriptomics (RNA), metaproteomics (proteins), and metabolomics (metabolites), through which novel biomarkers such as microbes, genes, proteins, and metabolites could be identified, which might have the potential to be used for the rapid and accurate diagnosis of human diseases caused by microbiota dysbiosis.

Microbial community is a complex but integral part of our body (human ecosystem) that is tightly associated with our health and disease (Segata et al., 2013). In order to comprehensively and accurately understand the microbial communities and their interactions with the hosts, an integrated approach that combines multi-omics data is starting to be under active develop, rather than relying on any single omics method. However, it is inherently difficult to integrate multi-omics data, e.g., metagenomes, metatranscriptomes, metaproteomes, and metabolomes, for systematic analysis (Knight et al., 2018) because these data are largely heterogeneous and are sourced from different time scales. Due to the importance of metaomics in comprehensive understanding of microbiomes, the studies and tools for the integration of different multi-omics data sets are becoming increasingly available, which greatly facilitates the development and translational potential of the metaomics approach in the field of human microbiota. So far, many pilot studies, preliminary analyses and comprehensive researches have innovatively explored the metaomics approach in the dissection of microbial communities and its interplays with hosts (Darzi et al., 2015; Aguiar-Pulido et al., 2016; Valles-Colomer et al., 2016; Boeri et al., 2022). For example, Valles-Colomer et al. (2016) systematically reviewed the application of metaomics in the complex and multifactorial disease IBD, which revealed that the approach held great promise in providing insights into IDB, though the interpretation of the metaomics data at multiple levels were very challenging. Boeri et al. (2022) summarized the current advantages of using metaomics approach to study microbiota–host interactions in the understanding of epilepsy with focuses on sample collection, extraction, and data processing, which could help in recognizing molecular pathways and biomarkers for microbiota–epilepsy connection, leading to development of novel clinical diagnostic methods. In addition, since the metaomics approach is data-intensive, many computational tools have also been developed and pipelines constructed for the comparative metaomics analysis so as to decipher the adaptations of microbial communities and microbiota–host interactions (Segata et al., 2013; Zhai et al., 2017; Sequeira et al., 2019). However, more computational tools are needed in this field in order to overcome the challenges of diversity and heterogeneity during the integration of the metaomics data. The phenotypes of complex microbial communities are constantly shaped by the dynamic interactions between hosts and their associated microbiota. In order to explore the full extent of microbial functions during the process, optimal, and efficient integration of multi-omics data derived from metagenomics, metatranscriptomics, metaproteomics, and metabolomics is essential, which significantly improves our knowledge of the human microbiome and its specific roles in the health and disease states of human beings. This is the reason why it is necessary to provide a timely and updated perspective overview of this exciting field. For an illustrative summary of the integration of the four metaomics approaches, refer to Figure 2 below.

## Challenges of Metaomics Approaches

Microbiota has been extensively studied for the past two decades from environment like water and soil to human body sites such as gut and skin, etc. In clinical settings, many diseases that are directly linked with microbial infections such as pneumonia, gastritis and vaginosis have been known to be caused by the disturbance of normal microflora. However, many well-known diseases that were previously unexpected to be microbe-relevant were also shown to have tight associations with the dysbiosis of human microbiomes such as mental disorders, CVD and cancer, etc. (Elinav et al., 2019; Velmurugan et al., 2020; Xu and Yang, 2021). Therefore, thorough understanding of the dynamic changes of human microbiome at both pathological and healthy states will greatly facilitate the understanding of disease mechanisms and promote the discovery of novel biomarkers at different levels (DNA, RNA, protein, metabolite, and species) *via* metaomics approaches, which could significantly improve the diagnostic efficiency and accuracy of multiple diseases in clinical laboratories. However, both the standalone omics techniques and combined metaomics approaches still face many challenges for their routine uses and real-world applications.

### Drawbacks of Standalone Omics Methods

The routine deployment of mNGS in clinical settings involves sample collection, nucleic acid extraction, library preparation, sequencing, computational analysis, and clinical interpretation of the data (Chiu and Miller, 2019). During the implementation of the mNGS pipeline, multiple factors should be considered for increasing the accuracy of the clinical diagnosis, such as sample stability during collection and transportation, diagnostic cost, turnaround time, computational complexity of datasets, and patient privacy, etc. (Chiu and Miller, 2019). In addition, sequencing and data extraction biases should also be considered since next generation sequencing is very well known to be biases toward certain GC range (Browne et al., 2020), which could be solved with methodological optimizations. During clinical diagnosis, the final and desired result is unbiased detection and reporting of all pathogens in a clinical sample, which involves targeted sequence capture, specialized computational tools, and explicative result reports and so on (Dekker and Dulanto Chiang, 2020). As for the metatranscriptomics, although it is complementary to metagenomics through dynamic characterization of microbiomes, some important restrictions should be pointed out in order to enhance the reproducibility and applicability of the approach, which may enable the integration of metatranscriptomic data into clinical settings (Bashiardes et al., 2016). Among these technical challenges, potential host RNA contamination and the short half-life of mRNA in the sample have been proven to be problematic (Bashiardes et al., 2016), which should be carefully handled during sample collection and RNA extraction. In fact, the procedures of RNA isolation, processing, sequencing, and analysis should be standardized so as to integrate the data into microbiome research. In addition, the metatranscriptomic data involves large-scale expression of genes, the discovery of which should be also validated *via* conventional diagnostic methods such as quantitative polymerase chain reaction (qPCR).

However, it is well known that the presence of DNA (metagenome) and mRNA (metatranscriptome) does not guarantee the presence of proteins and protein activities, not even mentioning the bioactive metabolites. In fact, different from metagenomics and metatranscriptomics, both metaproteomics and metabolomics are considered as functional tools to characterize microbial activities involving healthy and pathological states in human beings (Zhu et al., 2021). Therefore, metaomics pipeline integrating different omics approaches is necessary to generate a holistic view of clinical samples, which is also why metaproteomics and metabolomics are needed for sample analysis. Although sample preparation protocols for liquid chromatography-mass spectrometry (LC-MS) that were used for metaproteomics and metabolomics are becoming standardized, real-world analyses of these data are still facing many difficulties. For example, both metaproteomics and metabolomics experience lack of standardized protocols for sample preparations, inaccuracy of MS to measure low-concentration molecules, high costs of data generation and sophisticated downstream data analyses (Nyholm et al., 2020). In addition, the LC-MS approach is also limited to both insufficiency of reference database and inadequacy of normalization procedures (Ejigu et al., 2013; Vinaixa et al., 2016). Therefore, during the analysis of human microbiomes, the procedures should be scrutinized for best practice and the results should be carefully interpreted for accuracy.

### Limitations of Integrated Metaomics

It is true that metaomics has many advantages for microbial studies and holds the promise to revolutionize clinical diagnosis in foreseeable future. In fact, some of metaomics approaches have already been implemented in clinical diagnosis for certain circumstances such as precision medicine for drug-resistant tuberculosis (Leong et al., 2018) and identification of bacterial pathogens directly from clinical urine samples (Schmidt et al., 2017), etc. In addition, metaomics approach has also been applied to study complex disease such as epilepsy and IBD in order to understand the functions of microbiota in these diseases (Valles-Colomer et al., 2016; Boeri et al., 2022). Since metaomics is intrinsically a data-intensive field, well-trained personnel should also be a part of clinical diagnosis team during the coming metaomics era. Previously, restrictions to the use of metaomics such as low standardization of sample preparations and high costs of experiments have gradually been overcome, though there is still a gap that needs to be filled before the approach could be applied in real-world clinical settings. In addition to all the experimental procedures, novel and efficient computational tools are also essential for the application of metaomics, especially for data heterogenicity between and data integration across metagenomic, metatranscriptomic, metaproteomic, and metabolomic data sets, not even mentioning other more specialized omics techniques such as glycomics and lipidomics, etc. (Blackburn and Martens, 2016; Wang, 2022). Moreover, the development of pipelines to integrate standalone omics data, together with the equipment of sufficient computational storage space are also necessary for fast and efficient analysis during integration of metaomics data (Segata et al., 2013). Considering the complexity of the metaomics dataset, machine learning algorithms also provided a promising strategy to explore the microbiota-host interactions (Yuan et al., 2021). Taken together, in order to achieve a holistic analysis of microbiome and facilitate its diagnostic application in clinical settings, both experimental procedures and computational approaches should be enhanced and integrated to form a network-based approach in order to find true and reliable biomarkers for human diseases during clinical diagnosis.

## Conclusion and Perspectives

Although each omics approach provides valuable information separately for human microbiome analysis, it has been shown by various studies that these techniques could generate a more comprehensive picture for clinical diagnosis of diseases when combined together as metaomics. In fact, with the advancement of metaomics techniques in microbiome studies, many limitations for conventional clinical diagnosis could be overcome such as rapid recognition of unculturable pathogens, profiling of antibiotic resistance, causing pathogens of diseases, and harmful bioactive molecules, etc., which will greatly facilitate the efficient treatment and rapid management of microbial infections. In addition, supported by cumulative evidence of metaomics studies, it is gradually revealed that microbiota is indispensably involved in the basic biological activities of human beings through host-microbe interactions and the modulation of important human metabolic processes, while many studies have established the associations between human microbiomes and a variety of diseases such as obesity, diabetes mellitus, CVD and cancer, etc., though causative relationships between these associations still need further in-depth explorations. However, novel biomarkers from microbial perspectives, e.g., microbial compositions, gene levels, protein types, and metabolite concentrations, are still promising and hold the application potential in clinical settings. In this mini-review, we went through recent applications of standalone omics techniques and integrated metaomics in clinical setting, together with their current challenges, which reinforced the future of these novel methods in rapid and accurate disease diagnosis of human diseases.

## Author Contributions

LW, QZ, and ZM conceived and designed the framework of the study and provided the platform and resources. LW and QZ were responsible for project administration. LW, FL, BG, PQ, QL, JW, and SC carried out the literature review and performed the data analysis and visualization. All authors wrote and approved the final manuscript.

## Conflict of Interest

The authors declare that the research was conducted in the absence of any commercial or financial relationships that could be construed as a potential conflict of interest.

## Publisher’s Note

All claims expressed in this article are solely those of the authors and do not necessarily represent those of their affiliated organizations, or those of the publisher, the editors and the reviewers. Any product that may be evaluated in this article, or claim that may be made by its manufacturer, is not guaranteed or endorsed by the publisher.
